# Development of eosinophilic granulomatosis with polyangiitis during the clinical course of microscopic polyangiitis: A case report

**DOI:** 10.1097/MD.0000000000031401

**Published:** 2022-11-04

**Authors:** Hiroyuki Ide, Toshimasa Shimizu, Yuta Koike, Kuniko Abe, Kazuto Shigematsu, Shinya Nishihata, Kanako Kojima, Kunihiro Ichinose, Atsushi Kawakami

**Affiliations:** a Department of Immunology and Rheumatology, Nagasaki University Graduate School of Biomedical Sciences, Nagasaki, Japan; b Clinical Research Center, Nagasaki University Hospital, Nagasaki, Japan; c Department of Dermatology, Nagasaki University Graduate School of Biomedical Sciences, Nagasaki, Japan; d Department of Pathology, Japanese Red Cross Nagasaki Genbaku Hospital, Nagasaki, Japan.

**Keywords:** Eosinophilic granulomatosis with polyangiitis, microscopic polyangiitis, myeloperoxidase-antineutrophil cytoplasmic autoantibody, titanium implant, tocilizumab

## Abstract

Eosinophilic granulomatosis with polyangiitis (EGPA) is belongs to the antineutrophil cytoplasmic autoantibody (ANCA)-associated vasculitis (AAV) subgroups. EGPA, unlike other subgroups of AAV, including microscopic polyangiitis (MPA) and granulomatosis with polyangiitis, has the unique feature that both ANCA and eosinophilic inflammation are involved in its pathogenesis. Although AAV often relapses, there are currently no reports of EGPA developing during other subgroups of AAV. Herein, we document a case of EGPA that developed during the clinical course of MPA.

**Patient concerns:**

A 61-year-old Japanese woman was diagnosed with MPA based on interstitial lung disease and myeloperoxidase-ANCA positivity. After starting immunosuppression therapy, including prednisolone and tacrolimus, she was expected to achieve clinical remission. Nonetheless, she occasionally experienced MPA relapse, which required an increased prednisolone dose, rituximab, intravenous cyclophosphamide, and plasma exchange. Three years after MPA onset, she developed renal amyloidosis; thus, subcutaneous tocilizumab was added to her regimen. Following clinical remission, the administration interval of her subcutaneous tocilizumab therapy was extended and immunosuppressants were discontinued. She then developed bronchial asthma and mild eosinophilia (eosinophilic count: ~1000/μL). Further, a year later, she underwent total hip replacement using a titanium implant. Subsequently, she developed abnormal sensation in both hands, numbness, and muscle weakness, as well as palpable purpura and massive eosinophilia (eosinophilic count: ~8500/μL).

**Diagnosis:**

We diagnosed the patient with EGPA based on 5 items (asthma, multiple mononeuropathies, sinus abnormality, and extravascular eosinophils) of the 1990 American College of Rheumatology classification criteria.

**Interventions:**

We administered 400 mg/kg intravenous immunoglobulin for 5 consecutive days, 300 mg mepolizumab subcutaneously every 4 weeks, and 40 mg/day prednisolone following pulsed methylprednisolone therapy (1000 mg/day for 3 consecutive days).

**Outcomes:**

After these treatments, the patient’s symptoms improved, and eosinophilic count and inflammatory markers declined.

**Lessons:**

The present case suggests that EGPA can be induced by the development of eosinophilic inflammation in other subgroups of AAV.

## 1. Introduction

Antineutrophil cytoplasmic autoantibody (ANCA)-associated vasculitis is a small vessel necrotizing vasculitis, which has 3 subgroups including microscopic polyangiitis (MPA), granulomatosis with polyangiitis, and eosinophilic granulomatosis with polyangiitis (EGPA).^[1,2]^ Patients with MPA and granulomatosis with polyangiitis generally present with positive laboratory findings for ANCA, which plays a significant role in their pathogenesis.^[3]^ In contrast, the pathogenesis of EGPA is associated with ANCA as well as eosinophilic inflammation.^[4,5]^ Relapse after remission of ANCA-associated vasculitis usually develops within the same subgroups, even though the affected lesions may differ from those at the onset. Herein, we report a case in which EGPA developed during the clinical course of MPA.

## 2. Case report

**Figure 1.**
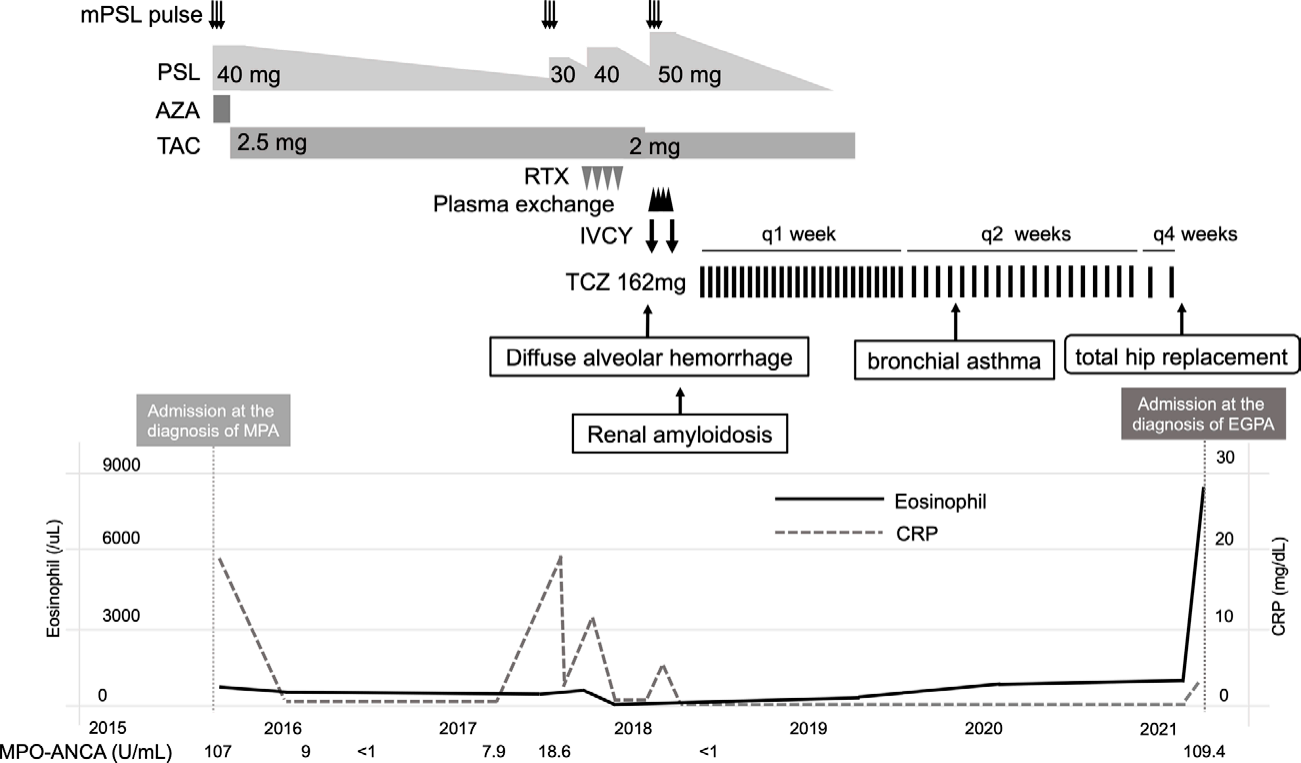
Clinical course. AZA = azathioprine, EGPA = eosinophilic granulomatosis with polyangiitis, IVCY = intravenous cyclophosphamide, MPA = microscopic polyangiitis, mPSL = methylprednisolone, MPO-ANCA = myeloperoxidase-antineutrophil cytoplasmic autoantibody, PSL = prednisolone, RTX = rituximab, TAC = tacrolimus, TCZ = tocilizumab.

A 61-year-old Japanese woman was admitted to our department with continuous fever and fatigue in November 2015. Laboratory tests showed elevated C-reactive protein (CRP; 19.18 mg/dL) and positivity for myeloperoxidase (MPO)-ANCA (107 U/mL) (Fig. 1). Thoracoabdominal computed tomography revealed bilateral reticular shadows and ground-glass opacities, which indicated interstitial lung disease (ILD). Thus, she was diagnosed with probable MPA based on 1 primary symptom (ILD) and positivity for MPO-ANCA as per the Ministry of Health, Labor and Welfare of Japan diagnosis criteria for MPA.^[6]^ Her blood eosinophil count was not elevated.

**Figure 2.**
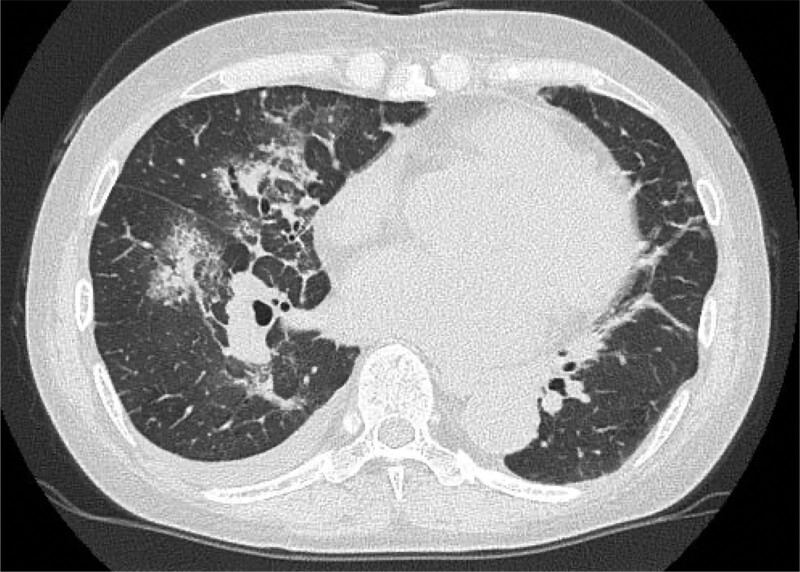
Thoracic CT imaging. Thoracic CT showing diffuse lung consolidation in the right lung. CT = computed tomography.

After being initiated on oral prednisolone (PSL; 40 mg/day) and azathioprine following pulsed methylprednisolone (mPSL) therapy (1000 mg/day for 3 consecutive days) in late November 2015, her symptoms improved. However, azathioprine was discontinued due to liver failure and replaced with tacrolimus (TAC) in late December 2015. Subsequently, she achieved clinical remission in March 2016, including no detection of MPO-ANCA while on PSL 20 mg/day and TAC 2.5 mg/day. Nonetheless, while taking PSL (7.5 mg/day) and TAC (2.5 mg/day) in May 2017, she was admitted to our department with abdominal pain as well as elevated CRP (19.58 mg/dL) and MPO-ANCA (18.6 U/mL), indicating a relapse in MPA. Thus, her PSL dose was increased to between 30–40 mg/day following pulsed mPSL therapy (1000 mg/day for 3 consecutive days). In addition, she was administered 4 doses of rituximab (375 mg/m^2^/week). Subsequently, her symptoms improved; however, her renal function gradually declined. Therefore, a renal biopsy was performed in January 2018. Before the renal biopsy results were back for analysis, she developed diffuse alveolar hemorrhage (Fig. 2), which was managed with 500 mg/body of intravenous cyclophosphamide monthly, plasma exchange, and an increase in PSL to 50 mg/day following pulsed mPSL therapy (1000 mg/day for 3 consecutive days; Fig. 1).

Subsequently, the renal biopsy revealed renal amyloidosis (amyloid A type) and no evidence of glomerulonephritis in February 2018. After the diffuse alveolar hemorrhage improved, she was started on 162 mg subcutaneous tocilizumab (TCZ) weekly for renal amyloidosis in April 2018. Following this, she achieved clinical remission of MPA. Consequently, dose tapering of PSL was initiated and discontinuation of the corticosteroid occurred in February 2019. In addition, TAC was discontinued in March 2019. Because of continued the remission, her TCZ administration interval was extended to every 2 weeks from July 2019 and then 4 weeks from November 2020. In December 2019, she developed bronchial asthma, which required inhaled corticosteroid/long-acting beta-agonist. Her serum eosinophilic count had remained approximately 1000/μL since this time.

**Figure 3.**
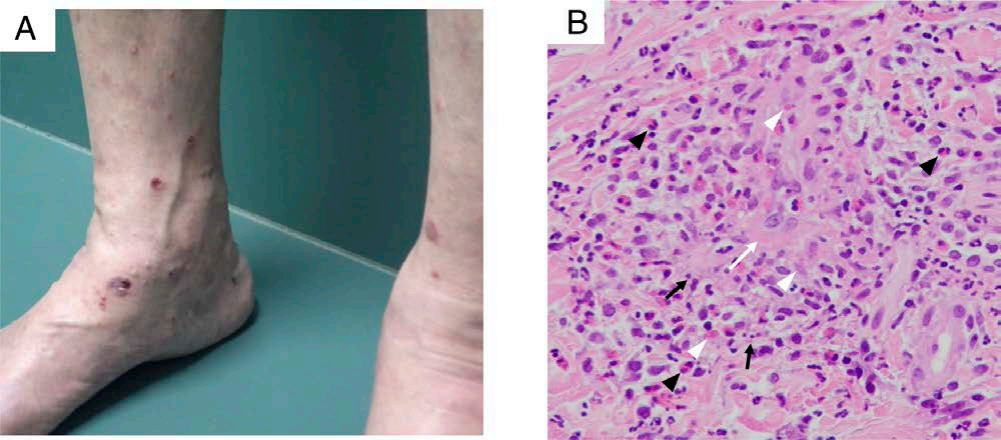
Skin findings. (A) Palpable purpura on both legs. (B) A skin biopsy specimen of the purpura on the left lower leg showing dermal extravascular leakage of red blood cells (white arrowhead), fibrinoid necrosis (white arrow), nuclear dust (black arrow), and eosinophil infiltration (black arrowhead). Hematoxylin and eosin staining × 200.

In early February 2021, she underwent right total hip replacement using a titanium implant for right hip osteoarthrosis at another hospital. Subsequently, she was admitted to our department presenting with abnormal sensation in both hands, numbness, and muscle weakness which had progressed within a few days in late March 2021. Physical examination revealed decreased grip strength (8.6 kg in the right hand and 0 kg in the left hand), sensory insensitivity in the ulnar nerve area of both hands, and loss of both biceps brachii and Achilles tendon reflexes. In addition, palpable purpura appeared on her forehead and both lower legs (Fig. 3A). No allergic reaction was noted around her right hip. Laboratory tests at the time of this admission revealed elevated white blood cell count (17800/μL), eosinophilic count (8500/μL), CRP (3.06 mg/dL), and MPO-ANCA (109.4 U/mL); however, her renal function was stable. Sinus and thoracic computed tomography revealed right maxillary sinusitis and no worsening of ILD. Head magnetic resonance imaging showed no evidence of a stroke. A nerve conduction study, conducted in her left upper and lower extremities, revealed multiple mononeuropathies of the axonal type. A skin biopsy specimen of the purpura on the left lower leg revealed nuclear dust, fibrinoid necrosis, eosinophil, neutrophil and macrophage infiltration surrounding the vessels, and extravascular leakage of red blood cells in the dermis, which indicated eosinophilic vasculitis (Fig. 3B). Bone marrow examination revealed no hematologic malignancy. In addition, no evidence of infection, including parasites, was confirmed.

We, thus, diagnosed the patient with EGPA based on 5 items of the 1990 American College of Rheumatology classification criteria.^[7]^ She was administered 400 mg/kg intravenous immunoglobulin for 5 consecutive days, 300 mg mepolizumab subcutaneously every 4 weeks, and 40 mg/day PSL following pulsed mPSL therapy (1000 mg/day for 3 consecutive days). After this regimen, inflammatory markers declined, and palpable purpura and nerve symptoms were relieved.

## 3. Discussion

MPA and EGPA are subgroups of ANCA-associated vasculitis classified as small vessel vasculitis in the 2012 revised Chapel Hill Consensus Conference Nomenclature of Vasculitides.^[1]^ The primary pathogenesis of MPA is an endothelial disorder caused by neutrophil activation due to ANCA binding, which leads to the release of reactive oxygen species and the formation of neutrophil extracellular traps.^[3]^ In contrast, the pathogenesis of EGPA is not only ANCA-mediated inflammation but also involves eosinophilic inflammation caused by eosinophil granule protein-induced cytotoxicity.^[4]^ Patients who develop EGPA have allergic predispositions such as bronchial asthma, resulting in T helper 2 (Th2) cells and group 2 innate lymphoid cells (ILC2) dominant responses.^[8–10]^ Th2 cytokines, released by Th2 cells and ILC2, especially interleukin (IL)-5, induce eosinophil activation, resulting in the release of eosinophil granule proteins, including major basic protein, eosinophil cationic protein, eosinophil peroxidase, and eosinophil-derived neurotoxin.^[5]^

On initial admission in 2015, the patient presented with fever, ILD, elevated MPO-ANCA, elevated CRP, and non-elevated blood eosinophil count, which were compatible with MPA. However, at the time of vasculitis relapse in 2021, her features were consistent with EGPA based on massive eosinophilia, multiple mononeuropathies, and eosinophil vasculitis in the purpura. In fact, using the new American College of Rheumatology/European Alliance of Associations for Rheumatology classification criteria for ANCA-associated vasculitis, the patient was classified as having MPA in 2015, and reclassified as having EGPA in 2021.^[11,12]^

Because the patient developed bronchial asthma in 2019, Th2- and ILC2-dominant inflammation was already thought to have occurred; however, it is not enough to cause EGPA. Therefore, we considered 2 factors as the ones that prompted the marked eosinophilic inflammation that led to the development of EGPA.

The first factor was IL-6 signal enhancement due to the extended administration interval of TCZ. IL-6 is an important inflammatory cytokine involved in the pathogenesis of allergic conditions, including asthma, via IL-6 downstream signaling.^[13]^ IL-6 signaling also prompts Th2 differentiation.^[14]^ In addition, a recent report described a case of EGPA which was triggered by extending the administration interval of TCZ in rheumatoid arthritis, suggesting that EGPA can be caused by IL-6 signal activation.^[15]^ To investigate a cytokine multiplex array of the patient’s serum, her serum IL-6 level at the development of EGPA was noted as 1076.8 pg/mL, which markedly increased compared to 55.34 pg/mL at MPA development (Table 1). We, thus, suggested that enhanced IL-6 signaling may lead to the development of EGPA.

**Table 1:** Serum cytokine levels in the presented case.

Variables	At MPA diagnosis	At EGPA diagnosis
IL-6	55.34	1076.8
IL-5	19.89	609.68
IL-13	61.41	96.84
CCL22	476.62	1449.15

CCL = C–C motif chemokine ligand, EGPA = eosinophilic granulomatosis with polyangiitis, IL = interleukin, MPA = microscopic polyangiitis. Units, pg/mL.

The second factor was a reaction to the titanium implants. Titanium implants have been reported to have less allergic response than other metal implants, with the patient presenting with no local allergic findings in this case. However, a previous in vitro study suggested that titanium activates macrophages, which promote the secretion of chemokine (C–C motif) ligands 17 and 22 (CCL17 and CCL22), inducing Th2 differentiation.^[16]^ In serum cytokines measured by cytokine multiplex array, the patient’s serum CCL22 level at the development of EGPA increased compared to that at the development of MPA. In addition, serum levels of IL-5 and IL-13 were elevated at the development of EGPA (Table 1). These findings suggested that the titanium implant may have led to the secretion of Th2 cytokines, such as IL-5 and IL-13 upon CCL22 production, contributing to the development of EGPA.

In conclusion, we present a patient who developed EGPA during the clinical course of MPA. The clinical course of EGPA has 3 phases: allergic, eosinophilic, and vasculitis, with the development of vasculitis occurring after the allergic and eosinophilic phases.^[17]^ The patient had an atypical clinical course of EGPA, which was triggered by allergic and eosinophilic conditions after developing ANCA-associated vasculitis. Our findings suggest that EGPA can be induced by the development of eosinophilic inflammation during other subgroups of ANCA-associated vasculitis. Further case and functional studies should be required to clarify the differential and overlapping pathogenesis of EGPA and other subgroups of ANCA-associated vasculitis.

## Acknowledgements

We would like to thank Editage (www.editage.com) for English language editing.

## Author contributions

**Writing – original draft:** Hiroyuki Ide, Toshimasa Shimizu.

**Writing – review & editing:** Hiroyuki Ide, Toshimasa Shimizu, Yuta Koike, Kazuto Shigematsu, Shinya Nishihata, Kanako Kojima, Kunihiro Ichinose, Atsushi Kawakami.
